# Evaluation of Intrastromal Riboflavin Concentration in Human Corneas after Three Corneal Cross-Linking Imbibition Procedures: A Pilot Study

**DOI:** 10.1155/2015/794256

**Published:** 2015-12-29

**Authors:** Antonella Franch, Federica Birattari, Gloria Dal Mas, Zala Lužnik, Mohit Parekh, Stefano Ferrari, Diego Ponzin

**Affiliations:** ^1^Department of Ophthalmology, SS Giovanni e Paolo Hospital, Sestiere Castello 6777, 30122 Venice, Italy; ^2^Fondazione Banca degli Occhi del Veneto, c/o Padiglione G. Rama, Via Paccagnella 11, Zelarino, 30174 Venice, Italy; ^3^Eye Hospital, University Medical Centre, Grablovičeva Ulica 46, 1525 Ljubljana, Slovenia

## Abstract

*Purpose*. To compare stromal riboflavin concentration after three corneal cross-linking (CXL) imbibition procedures: standard (EpiOff), transepithelial corneal (EpiOn), and iontophoresis-assisted technique (Ionto) using 0.1% hypotonic riboflavin phosphate.* Methods*. Randomized open-label pilot clinical study. Twelve corneas/12 patients with advanced keratoconus were randomly divided into 4 groups for CXL (*n* = 3). The corneas underwent imbibition with standard riboflavin EpiOff and with enhanced riboflavin solution (RICROLIN+) EpiOff, EpiOn, and iontophoresis techniques. Thereafter, deep anterior lamellar keratectomy procedure was performed and the obtained debrided corneal tissues were frozen. The maximal intrastromal riboflavin concentration was measured by high-performance liquid chromatography/mass spectrometry (mcg/dg).* Results*. The mean stromal concentration of riboflavin was 2.02 ± 0.72 mcg/dg in EpiOff group, 4.33 ± 0.12 mcg/g in EpiOff-RICROLIN+ group, 0.63 ± 0.21 mcg/dg in EpiOn-RICROLIN+ group, and 1.15 ± 0.27 mcg/dg in iontophoresis RICROLIN+ group. A 7-fold decrease in intrastromal riboflavin concentration was observed comparing EpiOn-RICROLIN+ and EpiOff-RICROLIN+ groups.* Conclusion*. The present pilot study indicates that both transepithelial CXL techniques in combination with hypotonic enhanced riboflavin formulation (RICROLIN+) were still inferior to the standard CXL technique; however, larger clinical studies to further validate the results are needed and in progress.

## 1. Introduction

Keratoconus (KC) is a progressive, degenerative, noninflammatory corneal thinning disease due to changes in organization and the structure of stromal corneal collagen fibers [1]. The disease usually manifests in the second decade of life with a relatively high prevalence in general population (1 in 1750 in white Europeans aged 10–44 years) [2]. In around 20% of keratoconic eyes, corneal transplantation is needed to restore vision [2].

In 2003, Wollensak with colleagues introduced the first available treatment option to halt the disease progression by chemically modifying the collagen fibers and increasing the corneal biomechanical strength and stability [3]. With the utilization of ultraviolet (UV) A light and riboflavin as a photosensitizer, they induced covalent cross-link bonds between collagen fibers [3]. Since then, numerous clinical studies and publications showed the efficacy and safety of corneal collagen cross-linking (CXL) technique in slowing down or halting KC progression. Currently, CXL has become a standard, low-invasive, and safe treatment option for progressive KC and several variations of the standard epithelium-off technique have been sought to avoid corneal epithelial debridement and thus increasing patients' safety and comfort [1]. However, when corneal epithelium is not removed, riboflavin penetration into the corneal stroma is decreased and lowers the efficacy of the procedure [4]. Thus, different strategies such as different riboflavin formulations that would enhance its penetration through intact epithelium, as well as prolongation of imbibition time, were used to overcome this issue. However, imbibition-time prolongation exposes corneas to excessive dehydration and thinning. Furthermore, deeper penetration of riboflavin can lead to increased corneal endothelium susceptibility to UVA toxicity [5].

To shorten the imbibition time, a new cross-linking technique was recently introduced using iontophoresis-assisted riboflavin administration (I-CXL). Due to riboflavin's small molecular weight, water solubility, and negative charge, it is a good candidate for iontophoresis [6]. Recently, the first preliminary results of up to 1 year postoperatively were reported indicating the efficacy of I-CXL in stabilizing the progression of CK [7]. Furthermore, a new hypoosmolar charged riboflavin solution (RICROLIN+; SOOFT Italia S.p.A., Montegiorgio, Italy) dextran-free that uses EDTA 0,1% and trometamol 0,05% as enhancers was introduced to optimize riboflavin stromal penetration by iontophoresis with fewer side effects [4, 5].

However, no comparative clinical studies on riboflavin penetration efficacy comparing transepithelial techniques to standard epithelium-off technique and the new riboflavin solution (RICROLIN+) were conducted to date.

Thus, the present pilot study aimed at determining differences in riboflavin concentration in the corneal stroma after three types of imbibition procedures (standard EpiOff, EpiOn, and iontophoresis-assisted administration) of 0.1% enhanced riboflavin solution were compared to a standard procedure in human corneas excised during deep anterior lamellar keratoplasty (DALK) was performed.

## 2. Materials and Methods

### 2.1. Pilot Study Design

A randomized open-label pilot study was conducted at the ophthalmology department of the SS Giovanni e Paolo Hospital (Venice, Italy) between February and March 2014. Before enrollment, written informed consent was obtained from all patients in the clinical study. The aim of the pilot clinical trial was to compare the efficacy of riboflavin penetration into the corneal stroma using three corneal cross-linking imbibition techniques before replacing the anterior corneal tissue with a donor cornea using deep anterior lamellar keratoplasty due to advanced keratoconus.

The primary outcome measure was the maximum corneal stromal riboflavin concentration (mcg/dg).

### 2.2. Patient Selection

Twelve patients (twelve corneas) with advanced keratoconus were selected for corneal cross-linking procedure before undergoing anterior lamellar keratoplasty (DALK).

The indication for DALK was defined after clinical and keratotopographical progression of keratoconus was identified.

Each patient had a pre- and postoperative standard ophthalmological examination, which included uncorrected distance visual acuity (UDVA), corrected distance visual acuity (CDVA), refractometry, corneal topography, pachymetry, endothelial cell count and confocal microscopy using, and slit-lamp biomicroscopy.

Only patients aged between 18 and 60 years were included in the study. The upper age limit was set at 60 years due to the nature of the disease [8]. Exclusion criteria included a corneal thickness less than 400 *μ*m, corneal scarring, previous refractive or other corneal surgery, a history of chemical burns, severe infections, corneal stromal dystrophies, autoimmune disease, and wearing contact lens less than four weeks before enrollment.

### 2.3. Treatment

Before DALK was performed, 12 eligible patients were randomized into four treatment groups: EpiOff group (gold standard or control group), EpiOff-RICROLIN+ group, EpiOn-RICROLIN+ group, and Ionto-RICROLIN+ group (Table 1).

EpiOff was designed as a control group (gold standard group) and included three eyes (*n* = 3). Riboflavin imbibition was performed according to a conventional collagen cross-linking protocol [9]. In short, after topical anesthetic instillation, the central corneal epithelium was removed to a diameter of 9 mm using a soft spatula. Standard riboflavin 0.1% eye drops (Ricrolin; SOOFT Italia S.p.A., Montegiorgio, Italy) were applied to the denuded cornea 1 drop every 1 to 2 minutes for 15 minutes. In EpiOff-RICROLIN+ group, after conventional corneal epithelium removal three corneas (*n* = 3) received RICROLIN+ (a hypotonic 0.1% riboflavin solution with enhancers dextran-free [including edetate sodium, tromethamine, bihydrate sodium phosphate monobasic, and bihydrate sodium phosphate bibasic]; SOOFT Italia S.p.A.) 1 drop every 1 to 2 minutes for 15 minutes. In EpiOn and Ionto groups, corneas received riboflavin imbibition via a transepithelial approach. In EpiOn-RICROLIN+ group, three corneas (*n* = 3) received RICROLIN+ for 30 minutes, 1 drop every 1 to 2 minutes. Although the enhancers in RICROLIN+ were designed to enable better riboflavin penetration into the corneal stroma through intact corneal epithelium, a longer imbibition time was used as reported in previous protocols [9]. Three corneas Ionto-RICROLIN+ group were soaked with RICROLIN+ using an iontophoresis system (IONTOFOR-CXL; SOOFT Italia S.p.A.) that was previously reported by Mastropasqua et al. [9]. For iontophoresis, two electrodes that are connected to a power generator are needed. One electrode (positive electrode) is placed on the patient's forehead, whereas the main negative electrode is contained in a rubber ring 10 mm in diameter, which is applied to the corneal surface and ensues constant riboflavin solution administration during the procedure. Iontophoresis is performed using a 1 mA/min intensity for 5 minute.

After riboflavin soaking, deep anterior lamellar keratoplasty (DALK) was started replacing 80% of the anterior portion of the diseased cornea with a healthy anterior corneal transplant.

Immediately after surgical removal of the anterior stromal tissue, slices from the EpiOff groups were frozen, while in the EpiOn and Ionto group the intact epithelium was removed before freezing to prevent the epithelial effect on the riboflavin concentration measurement.

### 2.4. Riboflavin Concentration Measurement

The concentration of riboflavin in each corneal stromal tissue sample was determined by high-performance liquid chromatography/mass spectrometry (HPLC/MS) after homogenization (Ultraturrax) and extraction in 50 : 50 (v/v) acetonitrile : water. The extracts were then centrifuged for 15 minutes at 10,000 rpm at 4°C. The sample extracts were diluted 100-fold with the initial mobile phase (10 : 90 100 mM ammonium formate buffer solution pH 3.2 : acetonitrile), and an aliquot of 5 *μ*L was injected into the HPLC column (Kinetex C8, 50 × 2.1 mm, 2.6 *μ*m). Samples were then analyzed using the Agilent 6410 Triple Quadruple Mass Spectrometer (Triple Quad MS) with an Electrospray Ionization (ESI) source (Agilent Technologies, USA). All data were acquired employing Agilent 6410 Quantitative Analysis version B.01.03 analyst data processing software. The calibration curve was obtained with a standard stock solution of riboflavin dissolved in 50 : 50 (v/v) acetonitrile : water to obtain an exact final concentration of 1 *μ*g/mL. The target calibration range was 0.676–1.690 ng/mL (LOD was 0.676 ng/mL). The isocratic flow rate was set at 0.1 mL/min for the 3 min of duration of each assay. Riboflavin was ionized under positive ionization conditions. The predominant peak in the primary ESI spectra of riboflavin corresponds to the [M–H–H_2_O]^+^ ion at *m*/*z* 377, and a secondary peak was detected at m/z 243. Chromatograms were integrated and the calibration curves plotted as the peak area of riboflavin. The determination coefficient (*r* ^2^) was 0.999.

### 2.5. Statistical Analysis

**Figure 1:**
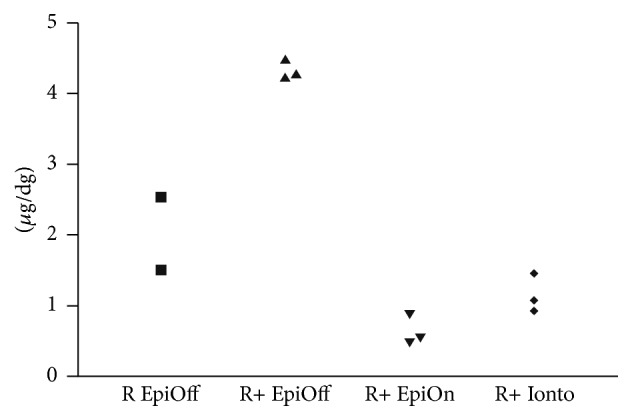
Scatter plot showing intrastromal riboflavin concentration (mcg/dg) per individual data in each group (EpiOff, EpiOff-RICROLIN+, EpiOn-RICROLIN+, and iontophoresis RICROLIN+). The pilot study results confirm that iontophoresis-assisted riboflavin imbibition reaches higher riboflavin intrastromal concentrations compared to EpiOn technique, but still the efficacy of riboflavin imbibition is inferior to EpiOff standard technique.

The limitations of our pilot study include the small sample size, which disabled a reliable statistical analysis. Thus, the results are presented as the mean riboflavin concentration ± SD. Individual data are plotted in a scatter plot (Figure 1). The preliminary pilot results comparing the difference of riboflavin concentration in the four treatment groups were tested by the Kruskal-Wallis nonparametric test coupled with Dunn's multiple comparison test.

## 3. Results and Discussion

### 3.1. Results

Recruitment for the pilot trial was completed after three months in 2013 with 12 eyes randomized into 4 treatment groups each containing 3 corneas, the EpiOff, EpiOff-RICROLIN+, EpiOn-RICROLIN+, and Ionto-RICROLIN+ groups. The groups were balanced regarding donor age and preoperative corneal thickness values. However, one cornea of the EpiOff treatment group was lost for analysis because of technical problems.

Overall, the mean riboflavin concentration in the excised anterior corneal slices after riboflavin imbibition using these three CXL techniques is reported in Table 2.

The mean stromal concentration of riboflavin was 2.02 ± 0.72 mcg/dg (*n* = 2) in the EpiOff group (gold standard group), 4.33 ± 0.12 mcg/dg in the EpiOff-RICROLIN+ group, 0.63 ± 0.21 mcg/dg in the EpiOn-RICROLIN+ group, and 1.15 ± 0.27 mcg/dg in the iontophoresis RICROLIN+ group. The pilot study results confirm that iontophoresis-assisted riboflavin imbibition reaches higher riboflavin intrastromal concentrations compared to EpiOn technique, but still the efficacy of riboflavin imbibition is inferior to EpiOff standard technique.

However, due to the small pilot sample size and due to an unexpected high variability between the two samples treated with standard riboflavin and the EpiOff procedure, a reliable statistical analysis could not be performed, as higher numbers of patients are required.

Nevertheless, in the EpiOff-RICROLIN+ group a much better and consistent intrastromal riboflavin concentration was noticed. Furthermore, a 7-fold increase in intrastromal riboflavin concentration was observed comparing the EpiOff-RICROLIN+ group and the EpiOn-RICROLIN+ group (4.33 ± 0.12 mcg/dg and 0.63 ± 0.21 mcg/dg, resp.) (Figure 1). Thus, these pilot study results indicate that the increase in imbibition time (from 15 minutes to 30 minutes) could not overcome the decrease of riboflavin penetration due to intact epithelium. On the contrary, in the iontophoresis group the corneal epithelium-dependent inhibition of riboflavin penetration could be partially overcome with iontophoresis-assisted technique even though the imbibition time was reduced to 5 minute, if compared to the standard EpiOff group.

### 3.2. Discussion

After a decade of clinical and experimental studies, it is well known that sufficient concentration of riboflavin in corneal stroma is crucial to obtain a biomechanical effect of corneal CXL [4, 9]. Thus, since riboflavin cannot penetrate intact corneal epithelium due to its chemical properties, the central corneal epithelium is mechanically debrided in current standard CXL (EpiOff) techniques to allow sufficient riboflavin stromal imbibition [5]. However, epithelial removal causes various side effects, including intra- and postoperative pain as well as visual decline in the first three months after procedure. Furthermore, it can predispose patients to serious corneal infections and loss of corneal transparency due to abnormal corneal stromal scarring processes [5]. Thus, in recent years much effort has been put into the development of new efficient transepithelial riboflavin penetration techniques. Different strategies were studied to enhance transepithelial riboflavin penetration, such as increasing riboflavin imbibition time and new riboflavin solution formulations to facilitate its transepithelial penetration. For this reason, several enhancers were proposed, with Ricrolin TE (SOOFT, Montegiorgio, FM, Italy) formulation being the most studied one with no consensus regarding studied clinical effectiveness in comparison to the standard CXL technique [5]. Another strategy to enhance riboflavin penetration through intact corneal epithelium that was recently introduced is the iontophoresis technique [10]. This is a noninvasive technique, which enables the transfer of charged small molecules throughout the tissues by means of a low intensity electric field [11]. The rate at which the charged molecules can penetrate into the tissue can be additionally increased with changing the characteristics of the solution as was done in the case of riboflavin solution. A special hypotonic riboflavin solution formulation (RICROLIN+) was developed for iontophoretic drug delivery to optimize riboflavin imbibition through intact corneal epithelium. This further enables shortening the treatment time as was recently shown in a 5-minute iontophoresis imbibition technique [9]. Moreover, a recent study by Mastropasqua et al. already compared the transepithelial (EpiOn and Ionto) techniques with the standard EpiOff technique for riboflavin stromal penetration, which showed a greater and deeper penetration rate of riboflavin when the iontophoresis-assisted transepithelial technique was used; however, the riboflavin concentrations were still lower than achieved with the standard EpiOff technique. Additionally, the study was done on human cadaver corneas not suitable for transplantation [9]. To date, no clinical comparative study evaluating these three CXL imbibition techniques was done on advanced keratoconus eyes.

Thus, in our recent pilot study we wanted to compare these three types of riboflavin imbibition procedures using a hypotonic 0.1% enhanced riboflavin solution formulation (RICROLIN+) with the standard CXL procedure in advanced keratoconic eyes immediately before deep anterior lamellar keratoplasty (DALK) was performed. Due to previous reports that showed no statistically significant differences in the intermediate and posterior corneal stromal riboflavin concentration among these 3 tested CXL techniques [4] and according to the fact that with DALK 80% of the diseased corneal tissue is removed, we could obtain enough corneal stromal material for further riboflavin concentration analysis using HPLC. Furthermore, to avoid the effect of the corneal epithelium in the transepithelial CXL group, the corneas were deepithelized before freezing. However, due to the small pilot sample size, the current pilot study is limited with reliable statistical analysis of the obtained results. Thus, although our preliminary pilot study data show no statistically significant difference in the mean stromal riboflavin concentration observed between the transepithelial techniques and the standard CXL technique, this might be due to an unexpected high variability between the two tested samples in the standard control group; therefore, the results were interpreted with caution and a larger number clinical study will be needed.

As expected, the lowest riboflavin penetration was observed in the passive EpiOn-RICROLIN+ and a slightly better permeability was observed in the iontophoresis group, reaching approximately 50% of the intrastromal concentration result of the standard EpiOff group. Importantly, the highest riboflavin concentrations were still found in the EpiOff group, with the pilot study results being similar to previously reported studies [4]. In fact, when we compare the EpiOn-RICROLIN+ group with the EpiOff-RICROLIN+ group, we observe a 7-fold decrease in intrastromal riboflavin concentration. These results demonstrate that also in our pilot study the increase in imbibition time (from 15 minutes to 30 minutes) could not overcome the barrier function of the intact corneal epithelium on riboflavin penetration, despite using the enhanced riboflavin solution formulation [4]. A recent study on rabbit eyes already showed that transepithelial riboflavin permeability was increased when using enhancers such as 0.01% benzalkonium chloride and 0.44% NaCl dextran-free; in our pilot study, we also demonstrated that with iontophoresis-assisted technique the results were better when compared to the EpiOn group but still inferior to the deepithelized CXL technique. The pilot study results might indicate that the corneal epithelium-dependent inhibition of riboflavin penetration might be at least partially successfully overcome with iontophoresis-assisted technique, if hypotonic enhanced riboflavin formulation was used, even though the imbibition time was reduced to 5 minutes. However, our encouraging pilot study results should be further evaluated in larger clinical studies that are already in progress.

Interestingly, we additionally observed a much better and consistent permeation in the EpiOff-RICROLIN+ group compared to the standard riboflavin group, indicating that further improvements (e.g., higher intrastromal riboflavin concentrations or shorter time treatments) could also be achieved in the EpiOff gold standard technique group.

## 4. Conclusions

In conclusion, the present pilot study indicates that both transepithelial CXL techniques in combination with hypotonic enhanced riboflavin formulation (RICROLIN+) were still inferior to the standard CXL technique. Furthermore, the highest riboflavin concentration was observed in the EpiOff-RICROLIN+ group, indicating that further improvements could also be achieved in the EpiOff gold standard technique group. Additionally, we demonstrated that prolongation of imbibition time yields an insufficient increase in the passive riboflavin penetration group (in the EpiOn group). On the contrary, if riboflavin is given by iontophoresis-assistance, a shortening of the imbibition time from 30 minutes to 5 minutes can be observed. Although, due to the small sample size, a reliable statistical analysis was not possible, these pilot study results are in accordance with previously reported data [4, 9] in human donor corneas. However, additional larger clinical studies are needed to further validate these results and the long-term efficacy of transepithelial CXL compared to the standard CXL protocol.

## Figures and Tables

**Table 1:** Tested groups. Both EpiOff groups had a 15-minute imbibition with Ricrolin (standard) or RICROLIN+. The EpiOn group had a 30-minute imbibition and the Iontophoresis group had a 5-minute imbibition with RICROLIN+.

	EpiOff	EpiOff-RICROLIN+	EpiOn-RICROLIN+	Ionto-RICROLIN+
Number of corneas analyzed	2	3	3	3
Impregnation	Ricrolin	RICROLIN+	RICROLIN+	RICROLIN+
Impregnation time	15 min	15 min	30 min	5 min

**Table 2:** The mean intrastromal riboflavin concentration of each treatment group determined by HPLC/MS after three different imbibition techniques were used.

Treated group	EpiOff	EpiOff RICROLIN+	EpiOn RICROLIN+	Iontophoresis RICROLIN+
Mean (mcg/dg)	2.02	4.33	0.63	1.15
SD (mcg/dg)	0.72	0.12	0.21	0.27

HPLC/MS: high-performance liquid chromatography/mass spectrometry; SD: standard deviation.
